# Executive Functions and Adaptive Behaviour in Autism Spectrum Disorders with and without Intellectual Disability

**DOI:** 10.1155/2014/941809

**Published:** 2014-01-21

**Authors:** Simonetta Panerai, Domenica Tasca, Raffaele Ferri, Valentina Genitori D'Arrigo, Maurizio Elia

**Affiliations:** ^1^IRCCS Oasi Maria SS., Via Conte Ruggero 73, 94018 Troina, Italy; ^2^Studio di Psicologia e Pedagogia, Via Giacomo Leopardi 23, 95127 Catania, Italy

## Abstract

Executive functions (EF) in autism spectrum disorders (ASD) have been often investigated, although results seem to be rather inconsistent. The first aim of this study was to detect which EF components are common to the ASD continuum (from high- to low-functioning ASD) and identify a possible EF profile for ASD people. Planning, mental flexibility, inhibition of response, generativity, and ecologic EF were investigated. This study was extended not only to high-functioning ASD, but also to ASD with intellectual disability (ID). The second aim was to find EF aspects correlating with adaptive skills in ASD. A total of 61 children participated in the study (27 ASD with and without ID and 34 controls). Results highlight an executive profile characterised by impaired flexibility and deficient planning; these deficits are associated with decreased adaptive ability, particularly socialization, and a deficient shifting in ecologic conditions. These features are present in all ASD subgroups with and without ID; for this reason, they might be assumed as being specific features in ASD.

## 1. Introduction 

The term “executive functions” (EF) refers to higher cognitive processes, mainly regulated by frontal lobes, which operate in daily complex situations and unusual contexts [1, 2]. EF include planning skills, working memory, mental flexibility, response initiation, response inhibition, impulse control, and action monitoring [3]. A number of neuropsychological studies have correlated EF with neural activities in different regions of frontal lobes and other circuits involving posterior cortical, subcortical, and thalamic areas [3, 4].

Several developmental disorders present with deficient EF, including autism spectrum disorders (ASD). Damasio and Maurer [5] observed that persons with autism showed some behaviours similar to those of persons with frontal lobe damage, thus suggesting a relation to specific neurological damage. This conceptual link between behaviour and brain led to the development of the theory of executive dysfunction [6, 7], by means of which repetitive behaviours and restricted interests have been best explained [6, 8].

EF in ASD have been often investigated, attempting to isolate intact and impaired processes; however, results seem to be rather convincing for some aspects of EF, but rather inconsistent for others, insomuch as many doubts remain whether executive dysfunctions could represent a diagnostic marker of the autistic conditions.

In a review by Hill [6] a number of studies were divided into the following executive domains: planning, mental flexibility, inhibition, generativity, and self-monitoring. Planning is a critical part of goal-oriented behaviour; it embodies the ability to formulate actions in advance and to approach a task in an organized, strategic, and efficient manner; this requires the ability of looking ahead, identifying, implementing, and monitoring different options in order to orient the current situation towards a new objective. Planning is generally investigated using the Tower of London (ToL) [9]. ASD children, adolescents, and adults with normal IQ have been reported to be significantly impaired on planning tasks when compared to age- and/or IQ-matched controls (with both typical development and developmental disorders, including dyslexia, Tourette syndrome, and attention deficit hyperactivity disorder (ADHD)). Using a computerised version of ToL, Happé et al. [10] reported normal performances in children with ASD without intellectual disability (ID). Older children showed a less number of extramoves than younger children; therefore, authors suggested that planning might be related to maturation and level of overall intellectual functioning. The assumption that planning deficits were related more to IQ than to autistic characteristics is also reported in Mari et al. [11]. Mental flexibility is the ability to spontaneously shift to a different action or thought in response to situational changes. Mental flexibility is generally investigated using the Wisconsin Card Sorting Test (WCST) [12]. Increased perseverations found in persons with ASD [6] than in normally developing children and children with other neurodevelopmental disorders are generally attributed to shifting difficulties; this deficit is maintained over time [13]. Flexibility deficits are also present in daily life, when family routines undergo some changes. Review of the literature seems to reveal impairments in cognitive flexibility not only during ASD individuals' adolescence and adulthood but also at younger developmental levels [7]. However, some studies [3, 14, 15] reported no significant differences in perseveration errors between children with ASD and IQ-matched controls (with typical development and developmental language disorder). Geurts et al. [16] affirmed that no consistent evidence for cognitive inflexibility was found, because deficits were clearly reported only in those studies using the WCST, while results turned out to be inconsistent in others using different measures. Response inhibition appears not to be a uniform process. Inhibition commonly refers to the ability to suppress a dominant, automatic, or prepotent response, but it also entails interference, emotional, and motor control; on a classic inhibition test, the Stroop task [17], children and adolescents with autism are generally found as unimpaired [18]; however, the interference effect is reported in other tasks (e.g., Go-no-Go tasks) [19] and in some studies using a computerised version of Stroop test [3]; Russell et al. [18] postulated that rules of some EF tests appear to be arbitrary to ASD individuals and causing the observed difficulties. Christ et al. [20] suggested that some specific aspects of inhibitory control may be impaired in children with ASD, and that there might be a relation between response inhibition and sustained attention. Other studies suggest that response inhibition and working memory (WM) are interdependent, but investigations on WM are also inconsistent, since deficits were detected only in a few of the studies (see [7, 21]). Results from a study by Ozonoff and Strayer [22] lead to conclude that WM is not one of the most seriously impaired EF in autism. As far as generativity is concerned, impaired performances have been generally reported for individuals with ASD. Generativity is defined as the capacity to spontaneously generate novel ideas and behaviours and it is thought to be a cause of the lack of spontaneity and initiative in autism, poverty of speech and action, and apparent failure to engage in pretence [6]. Turner [23] reported a correlational link between a poor performance in fluency tasks [24, 25] and high levels of repetitive behaviour in daily life, suggesting that generativity deficits could hinder the ability of controlling, regulating, and modifying behaviours. Self-monitoring refers to the process that enables individuals to see themselves as the makers of the changes when facing perceptual inputs, actions, and mental episodes. Studies have provided mixed evidence on a specific deficit in self-monitoring in ASD when compared with matched control groups (children with moderate learning disability and normally developing children) (see [6]). Monitoring one's verbal output is required on tests of verbal fluency to prevent items repetition; only one study [3] investigated the frequency of words repetition in fluency tasks, reporting more perseverative responses in ASD than in control children.

An open field of investigation is the correlation between executive and adaptive functioning. In some studies emerging EF are thought to be one of the sources of the heterogeneity in autistic individuals' functional outcomes [24, 25]. Gilotty et al. [26] found an inverse correlation between adaptive functioning and initiative/working memory deficits; in particular, the Socialization and Communication domains of the Vineland Adaptive Behavior Scale (VABS) [27] negatively correlated with executive dysfunctions. The results by Landa and Goldberg [28], however, did not support the hypothesis that executive dysfunctions could cause social and language impairments in ASD.

In this study, components of EF described by Hill [6]—namely, planning, mental flexibility, response inhibition, and generativity—were investigated; because of its importance, the ecologic EF component was also added. The aim of the study was to detect which EF components were common to ASD continuum (from high- to low-functioning ASD) and identify a possible EF profile for ASD people. Therefore, unlike the majority of studies in the literature, the investigation was extended to ASD associated with ID. The basic hypothesis was that whenever EF deficits and points of strength are present in the ASD continuum as compared to the control groups, it is reasonable to assume that they might represent specific features of ASD. The second aim was to find which aspects of EF correlate with adaptive skills in ASD.

## 2. Method 

### 2.1. Participants

A total of 61 children participated in the study. The experimental group included 27 randomly recruited ASD individuals, diagnosed according to DSM-IV-TR criteria [29] by a multidisciplinary diagnostic team, working in a research and treatment centre specialized in ID and brain aging. Then, the experimental group was divided into three subgroups: 11 individuals with ASD with normal cognitive level (IQ within one SD below/above the average; IQs range from 85 to 111), in the paper referred to as high-functioning ASD (HF-ASD); 8 individuals with ASD associated with borderline intellectual functioning (ASD-BIF; IQ within 1 and 2 SDs below the average; IQs range from 79 to 84); 8 individuals with ASD associated with mild intellectual disability (ASD-MID; IQ 2 SDs below the average; IQs range from 54 to 68).

The control group (no-ASD), made of 35 individuals, was divided into three subgroups: 9 children with typical development (TD), 12 presenting with BIF (IQs range from 71 to 84), and 13 with MID (IQs range from 55 to 69). BIF and MID diagnoses were made according to DSM-IV-TR criteria by a multidisciplinary diagnostic team. TD children were recruited from local public schools. Since several intellectual tests were reported in case histories of patients, derived IQs couldnot be used for comparisons; therefore, considering that visuospatial abilities are stronger than verbal abilities in ASD, all individuals were administered the Italian version of the Raven's Coloured Progressive Matrices (CPM) [30] in order to obtain comparable cognitive scores. Experimental and control groups were matched on the basis of the CPM intellectual test scores, in addition to chronological age and gender. Written informed parental consent was obtained prior to testing. Ethical approval was granted by the local ethical committee.

The sample characteristics are shown in Table 1. No statistical differences (*t*-test) and no large effect sizes (Cohen's *d* test) were found between the whole ASD and no-ASD groups for any of the matching criteria; as far as the subgroups comparisons are concerned, only in the case of ASD-MID and MID a large effect size was found, thus indicating a risk of II type error and the need for a larger sample size. This risk has been taken into consideration while analyzing results obtained from EF comparisons between subgroups with ID.

### 2.2. Measures

The following testing instruments were used to assess the different domains of EF and adaptive functioning.

*(1) Planning*. Tower of London (ToL), version included in the BVN 5–11 (Italian neuropsychological assessment battery for children aged from 5 to 11 years) [31] or BVN 12–18 [32]; starting from a fixed arrangement of three coloured beads on pegs having different sizes, children were required to copy a bidimensional model by rearranging the beads according to preestablished rules and move numbers. Clock drawing test (CDT), version included in the Italian Brief Neuropsychological Evaluation (ENB) [33]; starting from a circle previously drawn on a sheet of paper, children were required to draw clock numbers and hands at “a quarter to three.”

*(2) Mental Flexibility*. Wisconsin Card Sorting Test (WCST), Italian version [34]; children were required to sort cards according to one out of three unspecified rules (colour, shape, and number) and feedback about the choice (correct/incorrect).

*(3) Response Inhibition*. Stroop test, Italian version [35]; given a page with names of colours, written in different type colours (the type colour and the name of the colour do not match), children were required to name the colour of the word type; Go-no-Go trial,from the frontal assessment Battery (FAB) [36]; a prearranged sequence of mixed stimuli (one/two hits) was presented to children, who were required to perform a motor response when the one-hit stimulus (go) was presented and to withhold their motor response when a two-hit stimulus (no-go) was presented.

*(4) Generativity.* Verbal fluency tasks (category and phonemic) from the BVN 5–11 or 12–18 [31, 32]; children were required to generate as many different items as possible in accordance with animal, fruit, colour, and town semantic categories (category fluency) and with C, S, P phonemes (phonemic fluency).

*(5) Ecological EF. BRIEF-Parents Form *[37]. This questionnaire enables the assessment of executive function behaviours at home; it includes eight scales, grouped into three indexes: behavioral regulation index (BRI; it represents the capability of changing the cognitive set and modulates emotions and behaviours through inhibitory control; BRI is made of inhibit, shift, and emotional control scales), metacognition Index (MI; it represents the ability to cognitively self-manage tasks and to monitor one's own performance; MI is made of initiate, plan, organize, self-monitor and working memory scales), and global executive composite (GEC; it is an overarching summary score that incorporates all of the BRIEF clinical scales).

*(6) Adaptive Functioning*. VABS, expanded interview [27], a semistructured interview format tapping on children's personal, communication, and social skills, which was administered to parents.

### 2.3. Procedures

Tests were administered by a clinical psychologist. Tasks were presented in a preestablished order in one single session. In a separate session, questionnaires and interviews were administered to parents.

### 2.4. Statistics

The comparison between groups was carried out using the Student's *t*-test, and results were corrected by Bonferroni's test; Bonferroni threshold was calculated by dividing the error probability (*P* = .05) by the number of tests used to investigate the EF (*n* = 8; *P* ≤ .00625) and the adaptive functions (subtests of VABS: *n* = 4; *P* ≤ .0125). However, because of the relatively limited number of subjects available and in order to rule out possible type II errors, we also calculated effect sizes using Cohen's *d* value. Cohen's *d* is defined as the difference between two means divided by their pooled standard deviation. According to Cohen, 0.2 is indicative of a small effect, 0.5 of a medium effect size and 0.8 of a large effect size.

The correlation between adaptive and executive functioning was evaluated by means of the analysis of covariance (ANCOVA), with the subgroup being used as a covariate, for the calculation of the partial correlation coefficients.

The whole ASD group was compared to the whole no-ASD group; subsequently, pairs of subgroups were compared by using the following procedure: HF-ASD was compared with TD; ASD-BIF and ASD-MID were compared to BIF and MID without autism, respectively.

## 3. Results 

Results from comparisons are reported in separate sections, one for each function investigated.

### 3.1. Planning

Table 2 shows means and standard deviations obtained from group performances at the CDT and the ToL. The whole ASD group performed significantly lower than the no-ASD group at ToL; this difference was also confirmed by the comparison between the three ASD subgroups and the no-ASD matched control subgroups, respectively.

At the CDT, a significant difference was found in comparing ASD and no-ASD groups; as far as the subgroups comparisons are concerned, a significant difference was found only between ASD-MID and MID subgroups; the difference between HF-ASD and TD was not significant, but Cohen's *d* value (.76) showed a nearly large effect size.

### 3.2. Inhibition

Results (Table 2) show no significant differences between the two groups at both Stroop test and Go-no-Go testing. Comparisons between pairs of subgroups showed a statistical difference between ASD-BIF and BIF in the Go-no-Go tasks. No statistically significant difference but a large effect size was found in the Stroop test between ASD-MID and MID (t/sec and errors) and between HF-ASD and TD (only errors).

### 3.3. Generativity

Results (Table 2) from the comparison between the whole ASD and no-ASD groups showed a significant difference in category fluency. All ASD subgroups showed lower mean scores than the no-ASD subgroups, but a significant difference was found only between ASD-MID and MID. In the case of comparison between ASD-BIF and BIF, no statistically significant difference was found but only a large effect size. With respect to the phonemic fluency ASD-BIF/BIF and ASD-MID/MID, comparisons showed a large effect size.

### 3.4. Flexibility

WCST results are shown in Table 3. The comparison between the whole ASD and no-ASD groups showed a significant difference in error percentages, perseverative responses, and perseverative errors. The comparison between HF-ASD and TD subgroups showed a significant difference in the percentage of errors and perseverative responses, whereas in the perseverative errors a large effect size was found; the comparison between ASD-BIF and BIF showed a significant difference in the error percentages, perseverative responses, and errors, whereas the comparison between ASD-MID and MID showed a statistically significant difference in the percentage of perseverative responses and errors. With regard to the category numbers, Bonferroni's adjusted *P* value was significant only in the case of ASD-BIF and BIF comparison, but in all the other comparisons a large effect size was found.

### 3.5. Ecologic EF

BRIEF results are shown in Table 4. Following the comparison with the control group, only the BRI differentiated ASD from no-ASD; the comparison between pairs of subgroups showed a significant difference in the case of HF-ASD compared with TD and a large effect size in the other comparisons. Among the subdomains of BRI, inhibition (capability of withholding one's own behaviour if necessary) and shifting (capability of adaptive changing based on the situation requirements) differentiated ASD from no-ASD, with *P* < .008 (Bonferroni adjusted *P* value), but the only significant deficient parameter, common to all the three ASD subgroups, was shifting (HF-ASD and ASD-MID: *P* < .008; ASD-BIF: *P* = .016). As for inhibition, comparisons between pairs of subgroups showed a statistically significant difference for HF-ASD compared with TD, while the other comparisons showed a large effect size (ASD-BIF versus BIF: *d* = 1.41; for ASD-MID versus MID: *d* = .82). Neither a statistically significant difference nor a large effect size was found in emotional control (capability of modulating one's own emotional response) in the comparison between the whole groups with and without ASD; nevertheless, in the comparison between HF-ASD and TD a statistically significant difference was found (*P* < .008).

In the MI, the whole groups with and without ASD did not differ from each other, whereas a statistically significant difference was found in the comparison between HF-ASD and TD. This difference was confirmed in the majority of MI scales, such as working memory (hold information in mind for the purpose of completing a task; *P* = .032), planning/organization (anticipate future events, set goals and actions; *P* < .008), and monitoring (assess performance during or after finishing a task; *P* < .008). A significant impairment was found in organization of materials for ASD-FIL compared with FIL, and a large effect size was found in working memory. For ASD-MID no statistically significant differences were found in comparison with MID in any scale but only a large effect size in monitoring.

### 3.6. Adaptive Functioning

ASD significantly differed from no-ASD either at the VABS Composite Scale or at socialization and daily life subdomains (Table 5). In all the three subgroups with ASD, a significant difference was found in the comparison with the corresponding control subgroups, both in the Composite Scale and the socialization subdomain (socialization scores appeared markedly lower in ASD than in control subgroups). A difference in daily life skills was found from the comparison between ASD-MID and MID. For the other pairs of subgroups comparisons, a large effect size was found. Neither a statistically significant difference nor a large effect size was found in the communication subdomain from the comparison between ASD and no-ASD; nevertheless, results showed a significant impairment in ASD-MID when compared with MID and a large effect size in HF-ASD when compared with TD and in ASD-BIF when compared with BIF.

### 3.7. Correlations between Adaptive Skills and EF

Results are shown in Table 6.

Adaptive functioning was positively correlated with the intellectual level (according to CPM results), planning (ToL and Clock test), and generativity (verbal fluencies), in both ASD and controls. The increase in adaptive skills corresponds to the progressive increase in above-mentioned EF. ASD and controls showed the same trend, in that no differences between *r* values were found. In ASD, adaptive skills also positively correlated with the response inhibition and the mental flexibility (WCST perseverative responses and errors), whereas in the control groups no correlation was found. The search for differences between ASD and no-ASD *r* values showed a *P* value close to the statistical significance with regard to perseverative errors.

## 4. Discussion 

**Figure 1:**
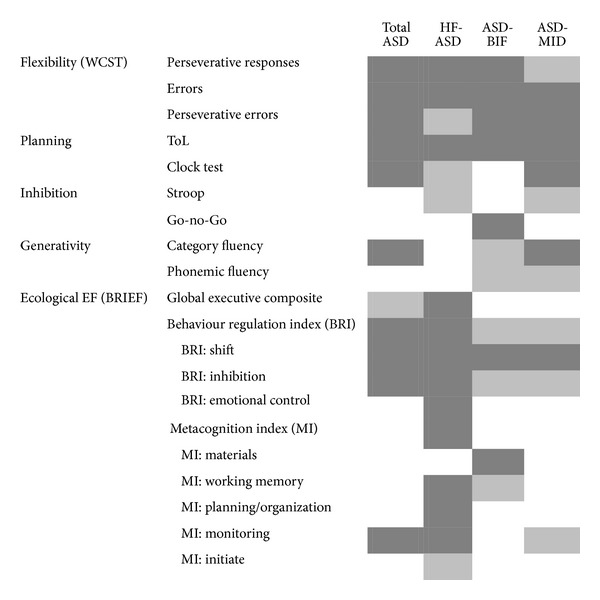
Graphic representation of EF impairments in ASD obtained in the study (dark grey: statistically significant impairment; light grey: nonsignificant impairment but large effect sizes).

In our study the EF spectrum (see Figure 1 for a graphic representation of results) appears to be not entirely impaired in ASD individuals.

Our results highlight an executive profile characterised by impaired flexibility and deficient planning of proper actions—especially in order to solve new problems—that requires the ability to virtually expect the effects of these actions; these deficits are associated with decreased adaptive abilities, particularly socialization, and a deficient behaviour regulation in ecologic conditions. These deficits are present in all ASD subgroups with and without ID; for this reason, they might be assumed as the typical feature of autistic disorders. On the other hand, the most preserved performances were found in inhibitory control tasks; generativity turned out to also be partially preserved, as well as metacognition and, from an adaptive viewpoint, VABS communication.

A number of characteristics differentiate the ASD subgroups, with and without ID, when compared with the relevant control groups: HF-ASD subgroup showed impaired performances even in structured cognitive tasks (particularly in planning and flexibility, whereas inhibition and generativity appeared to be more preserved) or in behavioural EF, with these latter appearing more impaired than the former; both ASD-BIF and ASD-MID showed an opposite tendency, with more preserved performance in ecological EF and a marked impairment in the EF structured tasks.

From a developmental viewpoint, immature inhibition is reported until 8 years of age in typical development and, in tasks like the Stroop one, some authors found consistent improvements in inhibition until adolescence and early adulthood; also, in flexibility and planning (tested with the WCST and ToL) improvements have been found up to early adolescence (see [38]). Tasks used for testing EF in our study were generally complex and it seems plausible to suggest that children with ASD are challenged by executive tasks because of their complexity. Nevertheless, significant differences between ASD and controls were not found in all these tasks; therefore, it remains unclear whether the task complexity might be an explanation for deficient executive performances in ASD.

In the following separate sessions, results obtained for each of the investigated EF will be discussed.

### 4.1. Planning

At the ToL, significant differences were found both in the whole sample and in the totality of subgroups. As for the CDT, the performance was impaired when considering the sample at a whole, whereas the subgroups showed remarkably differing levels of impairments, since only in the comparison between ASD-MID/MID subgroups, a statistically significant difference was found. Anyway, the weak results obtained from this test might be explained by the well-known marked difficulties in understanding the concept and the flow of time of ASD. Differing results obtained from ToL and CDT might be due to the fact that the two tests measure different domains of planning: the first requires the ability to look ahead [39] for determining the proper order of moves to successfully complete the task; in the second test, which basically requires praxis-constructive abilities, planning skills tap on laying out the numbers on the clock face through the recall of the clock mental image. In the literature, lower performance in ASD than in controls has been often reported when using the ToL test (see [3, 6, 19]). Several authors have assumed that these results can be related to the overall level of intellectual development and maturity [10]. Nevertheless, our data seem to disagree with such an assumption: although a correlation between ToL and CPM was found (*r* = .074, *P* < .001), performances of all subgroups, especially the HF-ASD, were significantly lower than those of controls, thus indicating that intellectual level is not the only factor that determines this gap. In our sample, the planning trial for the solution of a new problem appeared to be a specific characteristic of the ASD continuum.

CDT is generally used as a measure to investigate neuropsychological aspects of adults, in order to test visuospatial, graphomotor skills, and some domains of the EF. Its use in developmental age is not frequent in the literature. Kibby et al. [40] used the CDT with ADHD children who performed lower than the control group. The test correlated with the Wechsler Block Design, thus indicating its high sensitivity to test praxis-constructive skills. At Block Design, individuals with ASD without ID usually show excellent performances and, in some cases, even above the threshold. For this reason, the poor performance observed in our ASD sample is a little bit counterintuitive; on the other hand, the fact that impairments varied from one subgroup to another might likely be due to the most preserved praxis-constructive abilities. Unfortunately, no data have been reported in the literature that can be compared to our findings; however, CDT, being so short and simple, might be included in a battery for neuropsychological assessment during developmental age and might be a precious element to define specific profiles capable of differentiating several conditions, such as ADHD and ASD, having some overlapping characteristics.

### 4.2. Inhibition

Inhibition response seemed to be preserved in the whole sample; indeed no significant difference was found in the Stroop test (neither accuracy nor speed), although mean scores were lower in ASD than in controls. Nevertheless, results from subgroups seemed to be affected by the sample size, especially Stroop accuracy in HF-ASD and ASD-MID. Results are consistent with those from the literature which generally highlight similar performances in ASD and control groups for the inhibition of a prepotent response and control of interfering stimuli. The Stroop test is a complex cognitive task and some authors reported performance improvements until age 21 in typical development, thus indicating the gradual maturation of cognitive inhibition through adolescence and even early adulthood (see [38]), mainly consisting of refinements in speed and accuracy. Since similar performances have been found in ASD when compared to controls, data might suggest that executive dysfunctions in ASD cannot be explained on the basis of tasks complexity only.

Control of motor response also showed little differences in the mean scores in the comparison with the control group, but these differences were not statistically significant, except for the case of ASD-BIF. Our data are similar to those reported in the literature about ASD performance in Go-no-Go tasks.

### 4.3. Generativity

Only category fluency turned out to be impaired in the whole sample and, among the subgroups, only in ASD-MID. All ASD subgroups obtained lower mean scores, either in category or in phonemic fluency, but only in HF-ASD subgroup these abilities seemed to be preserved. The majority of studies in the literature report lower performances in ASD, in comparison with TD children. Results referring to HF-ASD subgroup could likely indicate a relation between this specific cognitive ability and the IQ level.

### 4.4. Flexibility

In our sample, the percentage of WCST perseverative responses and errors seemed to be significantly higher in ASD than in controls, irrespective of the IQ level. Consequently, the lack of flexibility, evident from increased perseverations, seemed to be a distinctive characteristic of our sample. This is probably due to the fact that a correct performance in WCST requires multiple skills, such as the production of rules, working memory, use of feedbacks provided by the experimenter, and shifting ability: all these skills are rather poor in ASD.

### 4.5. Ecologic EF

Only a few studies in the literature have used the BRIEF-Parent Form to test ecologic EF in ASD. Chan et al. [41], for example, examined the correlation between neurological functioning and EF; in that study, with a sample of HF-ASD subjects, all BRIEF indexes were higher than in controls. Our study confirms these results for HF-ASD subgroup, whereas for the whole sample, when compared with controls, a statistically significant difference was found only in the BRI and in some of its subscales, namely, shifting and inhibition, with impairments in shifting being common to all the ASD subgroups. Our findings draw a profile of ecologic EF mainly characterised by a decreased ability to adapt responses to different environmental conditions (shifting), irrespective of the cognitive level. These results confirm those obtained at the WCST in the current study.

### 4.6. Adaptive Functioning 

The composite VABS scale appeared significantly deficient in the whole ASD group and in all ASD subgroups, thus indicating serious adaptive deficits. Among them, the most marked and also specific characteristic was deficient socialization. Despite the lower mean scores, communication did not appear to be significantly impaired, except for the case of ASD-MID; nevertheless, for all the subgroups a large effect size was found. Although some doubts remain about the accuracy of these findings, more preserved abilities in communication than in the other VABS adaptive scales might be related to the fact that the scale detects present skills from a quantitative point of view and does not include items tapping on qualitative abnormalities, such as inferential ability, metaphorical comprehension, sense of humour, and prosody. Several studies have reported mixed language abilities, with preserved and impaired components [28]. Among the preserved components, phonological and grammatical domains, vocabulary, and lexical semantics are highlighted.

As far as daily living skills are concerned, only ASD-MID showed statistically significant differences in comparison with controls, but also in this case a large effect size was found in HF-ASD and ASD-BIF compared with relevant controls. Mean scores from these subgroups were lower than those from controls.

### 4.7. Correlation between Executive and Adaptive Functioning

The correlation analysis between VABS and the other tests on executive functions highlighted the presence of expected correlations which were similar in ASD and controls: therefore, the increase in adaptive skills was correlated to the increase in both cognitive level (CPM) and structured EF tasks (namely, ToL, CDT, category, and phonemic fluency). Neither in ASD nor in controls a correlation was found between VABS and BRIEF-GEC scores. This finding was not expected, but it is probably due to the fact that VABS and the other EF tests detect skills, while the BRIEF scale detects dysfunctions and executive problems. Our findings are in contrast with those of Gilotty et al. [26], who reported the presence of a negative correlation between communication and socialization, on one hand, and initiate and working memory dysfunctions on the other. In our ASD sample, none of the BRIEF indexes and subscales correlated with socialization and communication, whereas a negative correlation was found in controls between socialization and both GEC (*r* = −.4; *P* = .023) and MI (*r* = −.42; *P* = .016). Therefore, executive dysfunctions in daily life, especially in monitoring one's own behaviour, seem to be strongly associated with decreased socialization abilities in children without autism (with and without ID). Only in ASD sample, a negative correlation was found between VABS scores and WCST—perseverative responses and errors. Therefore, flexibility seemed an important factor that impact adaptive skills, especially communication and socialization. In our ASD sample, perseverations increased with the decreasing in adaptive skills, while this was not true for controls. First of all, it is important to keep in mind that perseverations of groups without autism were rather low; secondly, perseverations in controls might be differently interpreted from those in ASD individuals, and it is likely that the two groups activate differing processes to solve the same tasks. Perseverations could then be hypothesized as indicating the inability to “perceptually shift” from one kind of solution to another in autism but relating to the ability of monitoring the current activities in controls.

## 5. Conclusions 

EF are not a unitary process; they are best thought of as a set of multiple and distinct component processes. In this study some components of EF were investigated in ASD, in comparison with controls; deficient planning, flexibility, and behaviour regulation in ecological contexts seemed to be common to the ASD continuum (with and without ID); the EF profile of HF-ASD, when compared with controls, seemed characterised by either impaired or preserved EF in structured tasks, whereas a marked impairment was found in ecological situations; on the contrary, ASD-BIF and ASD-MID showed more preserved performances in ecological EF and marked impairments in the EF structured tasks.

EF and adaptive skills turned out to be correlated, even though it is not necessarily correct to assume that EF deficits are causative of social or communication impairments; indeed, deficits in social skills, such as joint attention, have an earlier development and would affect the development of skills that evolve later (e.g., EF).

This study has several limitations. The sample was relatively small; therefore, the research findings might be affected by the sample's size and the matching measures, weakening the results and conclusions; further studies with larger samples might confirm and better clarify data obtained, particularly about the points of weakness and strength in executive functioning of ASD. Another limitation of this study is the selection of executive tasks that were used; some executive functions, such as working memory and self-monitoring, were not investigated by means of structured tasks. A third limitation is that only one measure—perceptual reasoning—of cognitive level was used. Communication difficulties (especially in pragmatic communication) are a core feature of autism and have been indicated as a potential limiting factor on the EF development (see [24, 25]); therefore, children with ASD are less likely to use internal language in the service of executive control. The lack of verbal IQ in our study has prevented us to investigate how language and EF can be related to one another.

Another limitation is that the correlation between EF and ASD behavioural symptoms has not been performed: it might instead have been useful to understand whether EF deficits (and also which ones) represent a core feature of ASD. To this purpose, it would also be interesting to compare other disorders in which the same EF impairments are present (such as ADHD).

## Figures and Tables

**Table 1:** Characteristics of experimental (ASD) and control (no-ASD) groups.

	*N*	M/F	*P*	
Total ASD/no-ASD	27/34	24/5; 25/9	.76*	
HF-ASD/TD	11/9	9/2; 6/3	.96*	
ASD-BIF/BIF	8/12	6/2; 8/4	.52*	
ASD-MID/MID	8/13	7/1; 11/2	.78*	

	ASD Mean (SD)	No-ASD Mean (SD)	*P*	Cohen's *d*

	Chronological age			
Total ASD/no-ASD	9.82 (3.43)	11.32 (2.75)	.063**	−0.48
HF-ASD/TD	8.99 (3.08)	9.73 (2.62)	.97**	−0.26
ASD-BIF/BIF	9.56 (3.8)	11.86 (2.81)	.26**	−0.69
ASD-MID/MID	9.99 (4.48)	12.77 (2.51)	.16**	−0.77

	Raven's CPM			
Total ASD/no-ASD	21.59 (7.07)	22.15 (5.16)	.72**	−0.09
HF-ASD/TD	25 (6.27)	23 (5.14)	.87**	0.34
ASD-BIF/BIF	21.63 (4.5)	23.67 (3.06)	.24**	−0.53
ASD-MID/MID	14.63 (3.74)	17.92 (3.62)	.07**	−0.89

ASD: autism spectrum disorders; HF: high functioning; BIF: borderline intellectual functioning; MID: mild intellectual disability; TD: typical development; M/F: males/females; CPM: coloured progressive matrices; *chi square test; **t test.

**Table 2:** Means, SD, and statistically significant differences (*t* test; Bonferroni correction: *P* ≤ .00625; Cohen's test) obtained from ASD and no-ASD groups in planning, response inhibition, and generativity.

	ASD Mean (SD)	No-ASD Mean (SD)	*P*	Bonferroni adjusted *P* value	Cohen's *d*
	Planning: ToL				
Total ASD/no-ASD	4.3 (2.1)	6.6 (1.9)	<.001	<.008	−1.15
HF-ASD/TD	4.63 (2.02)	8 (1.73)	.003	.024	−1.79
ASD-BIF/BIF	4.5 (2.2)	7.33 (1.5)	.003	.024	−1.5
ASD-MID/MID	3 (1.69)	5 (1.08)	.004	.032	−1.41

	Planning: Clock drawing test				
Total ASD/no-ASD	3.3 (3.7)	6.2 (3.4)	.002	.016	−0.82
HF-ASD/TD	5.14 (4.36)	7.83 (2.42)	ns	ns	−0.76
ASD-BIF/BIF	4.6 (2.79)	5.96 (3.73)	ns	ns	−0.41
ASD-MID/MID	1.18 (1.69)	5.46 (3.42)	.005	.04	−1.55

	Response inhibition: *t*/sec Stroop word-colours				
Total ASD/no-ASD	89.8 (45.79)	90.7 (39.94)	.018	ns	−0.02
HF-ASD/TD	74.5 (20.83)	66.29 (10.21)	ns	ns	0.5
ASD-BIF/BIF	81 (32.37)	89.83 (45.45)	ns	ns	−0.22
ASD-MID/MID	146.6 (46.34)	107.18 (39.68)	ns	ns	0.91

	Response inhibition: errors Stroop word-colours				
Total ASD/no-ASD	4.85 (4.31)	3.41 (2.41)	ns	ns	0.41
HF-ASD/TD	3.33 (2.55)	1.71 (1.7)	ns	ns	0.75
ASD-BIF/BIF	4.5 (1.52)	3.42 (2.43)	ns	ns	0.53
ASD-MID/MID	9.4 (5.86)	5.09 (1.51)	.02	ns	1.01

	Response inhibition: Go-no-Go				
Total ASD/no-ASD	1.93 (1.07)	2.32 (0.98)	ns	ns	−0.38
HF-ASD/TD	2.38 (1.03)	2.78 (0.44)	ns	ns	−0.5
ASD-BIF/BIF	1.5 (0.93)	2.58 (0.51)	.003	.024	−1.44
ASD-MID/MID	1.75 (1.16)	1.77 (1.3)	ns	ns	−0.016

	Generativity: category fluency				
Total ASD/no-ASD	22.15 (18.97)	33.53 (11.83)	.006	.048	−0.72
HF-ASD/TD	34.5 (20.33)	43.11 (18.82)	ns	ns	−0.43
ASD-BIF/BIF	19.88 (14.62)	34.67 (10.08)	.015	ns	−1.18
ASD-MID/MID	7.63 (7.13)	25.85 (6.91)	<.001	<.008	−2.59

	Generativity: phonemic fluency				
Total ASD/no-ASD	13.22 (19.64)	14.5 (8.37)	ns	ns	−0.08
HF-ASD/TD	16.55 (12.12)	21.56 (10.19)	ns	ns	−0.44
ASD-BIF/BIF	8.38 (7.74)	15.75 (4.75)	.016	ns	−1.14
ASD-MID/MID	2.63 (3.66)	8.46 (4.96)	.01	ns	−1.34

ASD: autism spectrum disorders; HF: high functioning; BIF: borderline intellectual functioning; MID: mild intellectual disability; TD: typical development; ns: no significance.

**Table 3:** Means, SD, and statistically significant differences (*t* test; Bonferroni correction: *P* ≤ .00625; Cohen's test) obtained from ASD and no-ASD groups at WCST.

	ASD Mean (SD)	No-ASD Mean (SD)	*P*	Bonferroni adjusted *P* values	Cohen's *d*
	Flexibility: WCST errors %				
Total ASD/no-ASD	29.85 (9.38)	21.33 (7.7)	<.001	<.008	0.99
HF-ASD/TD	25.25 (6.53)	14.63 (4.5)	<.001	<.008	1.89
ASD-BIF/BIF	28.5 (7.15)	19.42 (4.48)	<.001	<.008	1.52
ASD-MID/MID	36.38 (10.57)	27.23 (7.51)	.031	ns	0.99

	Perseverative responses %				
Total ASD/no-ASD	20.89 (9.04)	10.67 (3.93)	<.001	<.008	1.47
HF-ASD/TD	19.38 (7.23)	8.75 (2.76)	<.001	<.008	1.94
ASD-BIF/BIF	19.63 (9.78)	10.42 (2.35)	.005	.04	1.29
ASD-MID/MID	24.63 (10.49)	12.08 (5.2)	.002	.016	1.51

	Perseverative errors %				
Total ASD/no-ASD	17.19 (8.62)	9.94 (3.45)	<.001	<.008	1.10
HF-ASD/TD	14.25 (6.2)	7.75 (1.83)	.01	ns	1.42
ASD-BIF/BIF	17 (7.54)	9.17 (2.25)	.003	.024	1.41
ASD-MID/MID	22.38 (10.61)	12 (4.1)	.005	.04	1.29

	Number of categories				
Total ASD/no-ASD	4.44 (1.85)	5.61 (1.03)	.02	ns	−0.78
HF-ASD/TD	4.75 (1.81)	6 (0)	ns	ns	−0.97
ASD-BIF/BIF	4.5 (1.69)	6 (0)	.006	.048	−1.25
ASD-MID/MID	3.5 (1.85)	5 (1.47)	.05	ns	−0.89

ASD: autism spectrum disorders; HF: high functioning; BIF: borderline intellectual functioning; MID: mild intellectual disability; TD: typical development; ns: no significance.

**Table 4:** Means, SD, and statistically significant differences (*t* test; Bonferroni correction: *P* ≤ .00625; Cohen's test) obtained from experimental and control groups at BRIEF-Parent Form.

	ASD Mean (SD)	No-ASD Mean (SD)	*P*	Bonferroni adjusted *P* value	Cohen's *d*
	BRIEF: behaviour regulation index				
Total ASD/no-ASD	59.59 (9.45)	44.65 (11.87)	<.001	<.008	1.39
HF-ASD/TD	58 (9.59)	34.78 (2.99)	<.001	<.008	3.26
ASD-BIF/BIF	60.63 (10.50)	47.25 (9.98)	.01	ns	1.3
ASD-MID/MID	60.63 (7.50)	49.08 (13.75)	.043	ns	1.04

	BRIEF: metacognition index				
Total ASD/no-ASD	84.63 (11.29)	83.68 (17.42)	ns	ns	0.06
HF-ASD/TD	82.5 (11.5)	59.22 (3.96)	<.001	<.008	2.7
ASD-BIF/BIF	85.13 (14.74)	94.58 (11.02)	ns	ns	−0.73
ASD-MID/MID	86.75 (7.52)	90.54 (9.72)	ns	ns	−0.44

	BRIEF: global executive composite				
Total ASD/no-ASD	143.44 (16.99)	125.5 (27.3)	.016	ns	0.78
HF-ASD/TD	140.38 (15.43)	94.00	<.001	<.008	4.11
ASD-BIF/BIF	145.75 (21.93)	141.83 (18.38)	ns	ns	0.19
ASD-MID/MID	147.38 (13.98)	140.08 (22.65)	ns	ns	0.38

ASD: autism spectrum disorders; HF: high functioning; BIF: borderline intellectual functioning; MID: mild intellectual disability; TD: typical development; ns: no significance.

**Table 5:** Means, SD, and statistically significant differences (*t* test; Bonferroni correction: *P* ≤ .0125; Cohen's test) obtained from experimental and control groups at VABS.

	ASD Mean (SD)	No-ASD Mean (SD)	*P*	Bonferroni adjusted *P* value	Cohen's *d*
	VABS: communication				
Total ASD/no-ASD	190 (49.55)	216.06 (45.7)	.041	ns	−0.54
HF-ASD/TD	210.27 (34.9)	238.67 (20.52)	.045	ns	−0.99
ASD-BIF/BIF	200.5 (27.52)	228.09 (17.27)	.015	ns	−1.20
ASD-MID/MID	146.14 (64.59)	206.08 (24.12)	.009	.036	−1.23

	VABS: daily life				
Total ASD/no-ASD	189.08 (72.09)	254.09 (67.55)	<.001	<.004	−0.93
HF-ASD/TD	204.91 (61.92)	279.22 (57.71)	.013	ns	−1.24
ASD-BIF/BIF	200.63 (72.27)	268 (54.14)	.032	ns	−1.05
ASD-MID/MID	151 (82.7)	243.67 (38.58)	.004	.016	−1.43

	VABS: socialization				
Total ASD/no-ASD	111.92 (34.85)	176.09 (50.68)	<.001	<.004	−1.47
HF-ASD/TD	118.09 (30.57)	214.67 (34.7)	<.001	<.004	−2.95
ASD-BIF/BIF	121.13 (24.2)	180.91 (32.98)	<.001	<.004	−2.07
ASD-MID/MID	91.71 (46.49)	157.42 (43.15)	<.002	<.008	−1.61

	VABS: Composite Scale				
Total ASD/no-ASD	604.38 (158.52)	741.97 (173.9)	<.001	<.004	−0.83
HF-ASD/TD	533.27 (117.75)	732.56 (111.19)	<.001	<.004	−1.74
ASD-BIF/BIF	522.25 (120.04)	677.01 (96.37)	<.006	<.024	−1.42
ASD-MID/MID	388.86 (187.5)	607.17 (88.36)	<.003	<.012	−1.49

ASD: autism spectrum disorders; HF: high functioning; BIF: borderline intellectual functioning; MID: mild intellectual disability; TD: typical development; ns: no significance.

**Table 6:** Correlations between adaptive and executive functioning in the experimental and control groups (ANCOVA—partial correlation coefficients) and *r* values comparison.

	ASD		Controls		Comparison *r* values
	*r*	*P*	*r*	*P*	*P*
VABS: CPM	0.72	.00007	0.54	.0026	ns
VABS: Tower of London	0.69	.00018	0.41	.025	ns
VABS: Clock test	0.62	.0013	0.59	.0007	ns
VABS: category fluency	0.49	.013	0.39	.038	ns
VABS: phonemic fluency	0.57	.0036	0.48	.0085	ns
VABS: Go-no-Go	0.43	.036	0.11	ns	ns
VABS: WCST perseverative responses %	−0.42	.039	−0.58	ns	ns (.08)
VABS: WCST perseverative errors %	−0.48	.019	−0.09	ns	ns (.06)
VABS: BRIEF global executive composite	0.005	ns	0.17	ns	ns
